# ARID1A IDR targets EWS-FLI1 condensates and finetunes chromatin remodeling

**DOI:** 10.1093/procel/pwae029

**Published:** 2024-05-18

**Authors:** Jingdong Xue, Siang Lv, Ming Yu, Yixuan Pan, Ningzhe Li, Xiang Xu, Qi Zhang, Mengyuan Peng, Fang Liu, Xuxu Sun, Yimin Lao, Yanhua Yao, Juan Song, Jun Wu, Bing Li

**Affiliations:** Department of Biochemistry and Molecular Cell Biology, Key Laboratory of Cell Differentiation and Apoptosis of Chinese Ministry of Education, Shanghai Key Laboratory for Tumor Microenvironment and Inflammation, Shanghai Jiao Tong University School of Medicine, Shanghai 200025, China; Department of Laboratory Medicine, Jiading Branch of Shanghai General Hospital, Shanghai Jiao Tong University School of Medicine, Shanghai 201803, China; Clinicopathological Diagnosis & Research Center, The Affiliated Hospital of Youjiang Medical University for Nationalities, Baise 533000, China; Key Laboratory of Tumor Molecular Pathology of Guangxi Higher Education Institutes, Baise 533000, China; Department of Biochemistry and Molecular Cell Biology, Key Laboratory of Cell Differentiation and Apoptosis of Chinese Ministry of Education, Shanghai Key Laboratory for Tumor Microenvironment and Inflammation, Shanghai Jiao Tong University School of Medicine, Shanghai 200025, China; Department of Biochemistry and Molecular Cell Biology, Key Laboratory of Cell Differentiation and Apoptosis of Chinese Ministry of Education, Shanghai Key Laboratory for Tumor Microenvironment and Inflammation, Shanghai Jiao Tong University School of Medicine, Shanghai 200025, China; Department of Biochemistry and Molecular Cell Biology, Key Laboratory of Cell Differentiation and Apoptosis of Chinese Ministry of Education, Shanghai Key Laboratory for Tumor Microenvironment and Inflammation, Shanghai Jiao Tong University School of Medicine, Shanghai 200025, China; Shanghai Institute of Hematology, State Key Laboratory of Medical Genomics, National Research Center for Translational Medicine at Shanghai, Ruijin Hospital Affiliated to Shanghai Jiao Tong University School of Medicine, School of Life Sciences & Biotechnology, Shanghai Jiao Tong University, Shanghai 200025, China; Department of Laboratory Medicine, Jiading Branch of Shanghai General Hospital, Shanghai Jiao Tong University School of Medicine, Shanghai 201803, China; Department of Biochemistry and Molecular Cell Biology, Key Laboratory of Cell Differentiation and Apoptosis of Chinese Ministry of Education, Shanghai Key Laboratory for Tumor Microenvironment and Inflammation, Shanghai Jiao Tong University School of Medicine, Shanghai 200025, China; Department of Biochemistry and Molecular Cell Biology, Key Laboratory of Cell Differentiation and Apoptosis of Chinese Ministry of Education, Shanghai Key Laboratory for Tumor Microenvironment and Inflammation, Shanghai Jiao Tong University School of Medicine, Shanghai 200025, China; Department of Biochemistry and Molecular Cell Biology, Key Laboratory of Cell Differentiation and Apoptosis of Chinese Ministry of Education, Shanghai Key Laboratory for Tumor Microenvironment and Inflammation, Shanghai Jiao Tong University School of Medicine, Shanghai 200025, China; Department of Biochemistry and Molecular Cell Biology, Key Laboratory of Cell Differentiation and Apoptosis of Chinese Ministry of Education, Shanghai Key Laboratory for Tumor Microenvironment and Inflammation, Shanghai Jiao Tong University School of Medicine, Shanghai 200025, China; Department of Biochemistry and Molecular Cell Biology, Key Laboratory of Cell Differentiation and Apoptosis of Chinese Ministry of Education, Shanghai Key Laboratory for Tumor Microenvironment and Inflammation, Shanghai Jiao Tong University School of Medicine, Shanghai 200025, China; Department of Biochemistry and Molecular Cell Biology, Key Laboratory of Cell Differentiation and Apoptosis of Chinese Ministry of Education, Shanghai Key Laboratory for Tumor Microenvironment and Inflammation, Shanghai Jiao Tong University School of Medicine, Shanghai 200025, China; Department of Biochemistry and Molecular Cell Biology, Key Laboratory of Cell Differentiation and Apoptosis of Chinese Ministry of Education, Shanghai Key Laboratory for Tumor Microenvironment and Inflammation, Shanghai Jiao Tong University School of Medicine, Shanghai 200025, China; Department of Laboratory Medicine, Jiading Branch of Shanghai General Hospital, Shanghai Jiao Tong University School of Medicine, Shanghai 201803, China; Clinicopathological Diagnosis & Research Center, The Affiliated Hospital of Youjiang Medical University for Nationalities, Baise 533000, China; Key Laboratory of Tumor Molecular Pathology of Guangxi Higher Education Institutes, Baise 533000, China; Department of Biochemistry and Molecular Cell Biology, Key Laboratory of Cell Differentiation and Apoptosis of Chinese Ministry of Education, Shanghai Key Laboratory for Tumor Microenvironment and Inflammation, Shanghai Jiao Tong University School of Medicine, Shanghai 200025, China

**Dear Editor,**

The human SWI/SNF chromatin remodeling complexes are essential for modulating genomic architecture and increasing DNA accessibility, thereby regulating gene transcription (Clapier et al., 2017). These complexes exhibit a high frequency of mutations across all tumors, with a mutation rate of 20% observed in the coding genes of 29 subunits (Kadoch et al., 2013). Mutations in several subunits are closely associated with tumor occurrence and development, underscoring the physiological and pathological significance of human SWI/SNF complexes in maintaining regular cellular functions and gene regulation (Kadoch et al., 2013).

The canonical BAF (cBAF) family of human SWI/SNF chromatin remodelers plays a critical role in the initiation and progression of various types of cancers (Wu and Roberts, 2013), including Ewing sarcoma. Ewing sarcoma is primarily driven by an oncogenic fusion protein EWS-FLI1. This fusion protein is the product of a chromosomal translocation event that fuses the 5ʹ end of the *EWSR1* gene with the 3ʹ end of the *FLI1* gene (Boulay et al., 2017). The chimeric transcription factor EWS-FLI1 undergoes liquid-liquid phase separation (LLPS) and binds to neomorphic microsatellite DNA regions, which in turn recruits cBAF to activate an oncogenic transcriptional program in Ewing sarcoma (Boulay et al., 2017). However, the precise mechanism by which phase-separated oncogenic transcription factors recruit cBAF to target genes remains unresolved.

To elucidate the specific subunit of cBAF that interacts with phase-separated EWS-FLI1, we implemented an unbiased screening approach that combines proximity labeling and mass spectrometry techniques (Wolf et al., 2022). We introduced a customized biotin ligase, TurboID, at the N-terminus of both the wild-type EWS-FLI1 and an LLPS-deficient mutant (EWS(YS)-FLI1), bearing 37 tyrosine-serine mutations (Boulay et al., 2017) (Fig. 1A). This experimental design allowed us to specifically identify proteins associated with EWS-FLI1 condensates, while excluding those associated with the free form of the protein. Lentiviral infection was performed, resulting in stable expression of these fusion proteins in HEK293T cells, which were confirmed through Western blot analysis (Fig. S1A). We optimized the purification step to augment the stringency of the assay (Fig. 1B) and efficiently eliminate other subunits present in assembled complexes (Fig. S1B). The purified samples underwent mass spectrometry analysis (Table S1), and the results were presented as a volcano plot in Fig. 1C. Compared to EWS(YS)-FLI1, EWS-FLI1 showed an increased association with 169 proteins (gained) and a decreased binding to 260 proteins (lost) (Fig. 1C). Subsequent Gene Ontology analysis of the gained proteins revealed a significant elevation in various categories related to transcriptional regulation and mRNA splicing processes, including transcription factors and chromatin remodeling complexes (Fig. S1C). Conversely, the lost proteins were predominantly enriched with functionally unrelated constituents such as cytoskeleton-related proteins. Notably, the proximity-labeled proteins unique to EWS-FLI1 included several subunits of the cBAF complex, such as ARID1A, BRG1, and BAF155. Among these, ARID1A within the cBAF complex exhibited the highest difference in abundance between EWS-FLI1 and EWS(YS)-FLI1, with a log2 fold change ratio of 4.78 (Fig. 1C).

**Figure 1.**
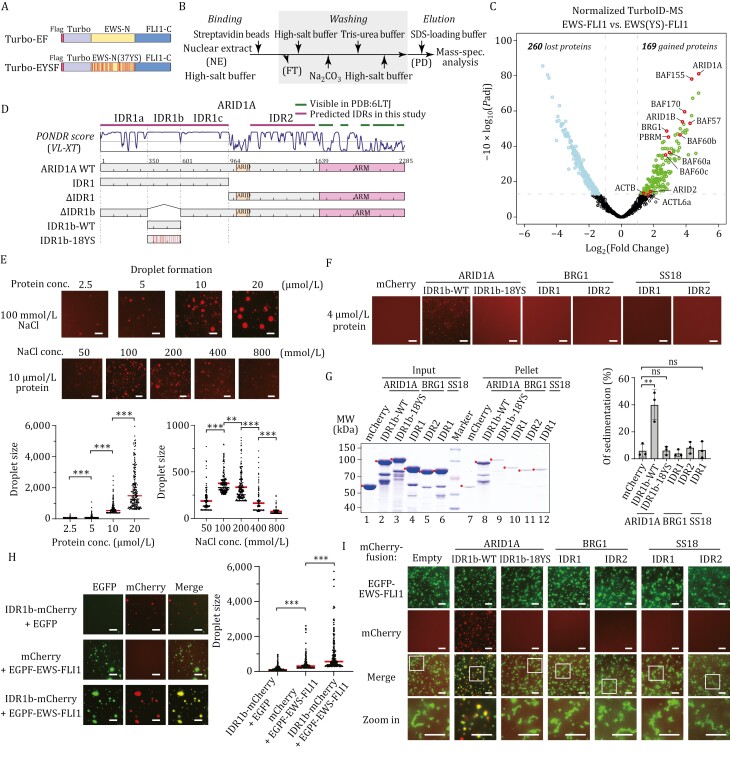
Phase-separated EWS-FLI1 recruits cBAF complex via ARID1A IDR1b. (A) Diagram showing EWS-FLI1 and EWS(YS)-FLI1 used in the TurboID assay. Turbo-EF represents TurboID-EWS-FLI1, while Turbo-EYSF represents TurboID-EWS(YS)-FLI1. (B) Workflow illustrating the enrichment of biotin-labeled proteins from HEK293T nuclear extract in a TurboID assay. NE: nuclear extract; PD: pull-down; FT: flow through. (C) Volcano plot from the TurboID assay displays the EWS-FLI1-specific proximally-labeled interactome compared to that of EWS(YS)-FLI1. Gained proteins (fold change—FC > 2.0, adjusted *P*-value—*P*adj < 0.05), lost proteins (FC < 0.5, *P*adj < 0.05) are labeled. Subunits of the human SWI/SNF complexes are highlighted in circle. (D) Predicted IDRs (top), PONDR (Predictor of Natural Disordered Regions) VL-XT score (middle) of ARID1A, and schematic of ARID1A truncations and mutations used in this study (bottom). (E) ARID1A IDR1b forms phase-separated condensate. Representative images (top) and quantification diagrams (bottom) of ARID1A IDR1b droplet formation with gradient protein concentration or NaCl concentration. The droplet formation reactions contain 8% PEG-8000. Scale bar, 10 µm. The largest 200 droplets in each group are presented in the quantification diagrams. The *P*-value is calculated by unpaired Student’s *t*-test. ** indicates *P*-value < 0.01, *** indicates *P*-value < 0.001. (F) Representative images of droplet formation of wild-type or mutated IDRs of subunits in the cBAF complex. The droplet formation reactions contain 8% PEG-8000. Scale bar, 10 µm. (G) Sedimentation assay (right) and quantification diagram (left) of the indicated IDRs. The sedimentation assay is presented on SDS-PAGE and stained with Coomassie blue. The final concentration of each protein in the reaction is 4 μmol/L. The quantification data comprise three individual replicates and are represented as mean ± SD. The *P*-value is calculated by unpaired Student’s *t*-test. “ns” indicates not significant, ** indicates *P*-value < 0.01. (H) Representative images and the quantification diagram of droplet colocalization of EGFP-tagged EWS-FLI1 and mCherry-tagged ARID1A IDR1b. Each protein’s final concentration in the reaction is 8 µmol/L, supplemented with 6% PEG-8000. Scale bar, 10 µm. The largest 200 droplets in each group are presented in the diagram. The *P*-value is calculated by unpaired Student’s *t*-test. *** indicates *P*-value < 0.001. (I) Representative images of droplet colocalization of EGFP-tagged EWS-FLI1 and mCherry-tagged IDRs of subunits in the cBAF complex. The final concentration of each protein in the reaction is 4 μmol/L. Images in white boxes are enlarged for detail and shown at the bottom as “Zoom In”. Scale bar, 10 µm.

ARID1A (AT-rich interacting domain containing protein 1A) is a crucial subunit within the cBAF complex, which plays an indispensable role in chromatin occupancy and remodeling activity in mammalian cells (He et al., 2020). Moreover, ARID1A exhibits the highest frequency of mutations among the subunits of human SWI/SNF complexes in cancers, and this mutation has been strongly associated with the occurrence and development of ovarian clear-cell carcinoma, colon cancer, and other types of tumors (Wu and Roberts, 2013). Previous studies have established that ARID1A interacts with transcription factors (Wu and Roberts, 2013). It has been observed that ARID1A plays a crucial role in recruiting the cBAF complex to enhancers by interacting with transcriptional activators and coactivators (Clapier et al., 2017). Additionally, ARID1A is known to participate in other biological processes, such as double-strand break and mismatch repair, through its interaction with key regulators (Clapier et al., 2017). Furthermore, the activator binding ability of Swi1, the yeast homolog of ARID1A, suggests a conserved targeting property of ARID1A during evolution (Clapier et al., 2017). Considering these findings in conjunction with our TurboID results, we speculated that ARID1A may serve as the primary interface between EWS-FLI1 and the cBAF complex.

To determine the specific regions of ARID1A that target the cBAF complex, we conducted an in-depth analysis combining structural information (He et al., 2020), amino acid composition, and intrinsic disorder predictions. Through this analysis, we identified intrinsically disordered regions (IDRs) and their corresponding subsegments within the major subunits of the cBAF complex (Fig. S1D, S1F, and S1G). Notably, ARID1A contains a long N-terminal IDR (IDR1) with a more diverse amino acid composition across different subsegments (Fig. 1D). It is worth noting that neither the cryo-EM structures of the human cBAF complex (He et al., 2020) nor the yeast SWI/SNF complex (Han et al., 2020) reveals any stable structure of the corresponding IDRs within ARID1A or Swi1. Considering that the liquid–liquid phase separation (LLPS) ability of transactivation domains in transcription factors has been shown to correlate with their gene activation potentials (Boija et al., 2018), we speculate that the IDRs within ARID1A contribute to co-activator recruitment by forming phase separation condensates.

Given the liquid–liquid phase separation of the N-terminal intrinsically disordered region (IDR) in EWS-FLI1, facilitated by multivalent interactions primarily mediated through the SYGQ quartet amino acid cluster (Boulay et al., 2017), we investigated the IDRs within cBAF, which also contain a high abundance of these residues. We observed that ARID1A contains two distinct IDR sections, as shown in Fig. 1D. IDR1 possesses a significant concentration of the SYGQ amino acid cluster, which can be further divided into three sections: IDR1a, IDR1b, and IDR1c. Remarkably, IDR1b contains 18 tyrosine residues, making it the most tyrosine-rich region within IDR1 (Figs. 1D and S1D). Thus, our initial investigation focused on determining whether IDR1b has the capability to undergo phase separation and form droplets. To this end, we expressed and purified IDR1b harboring an N-terminal GST tag and a C-terminal mCherry tag using a prokaryotic protein expression system (Fig. S1E). Upon purification, the IDR1b protein of ARID1A was subjected to conditions favorable for liquid–liquid phase separation (LLPS), resulting in the formation of distinct droplets. The emergence of these droplets exhibited a strong dependence on the concentrations of both the protein and the salt, aligning with the expected behavior of tyrosine-mediated phase separation (Fig. 1E). To further substantiate the LLPS behavior of ARID1A IDR1b, we performed droplet formation assays under a range of conditions. The experimental data indicated that the *in vitro* formation of ARID1A IDR1b droplets necessitates the presence of molecular crowding agents. Notably, droplet formation was successfully induced by various agents, such as PEG-8000, Dextran, and Ficoll (Fig. S1H). In contrast, in the absence of crowding agents, ARID1A IDR1b failed to form droplets, underscoring the importance of a moderately crowded cellular milieu for its phase separation. This finding implies that the intracellular environment, with its inherent crowded nature, may be essential for the LLPS of ARID1A IDR1b.

To confirm the role of tyrosines in the liquid–liquid phase separation (LLPS) capacity of IDR1b, we substituted all 18 tyrosines with serines, generating the mutant IDR1b-18YS. Subsequently, we performed LLPS experiments using the same parameters as above. Notably, our results revealed that under these conditions, IDR1b-18YS displayed no noticeable droplet formation compared to the wild-type IDR1b (Fig. 1F). Moreover, a sedimentation assay demonstrated a significant decrease in the sedimentation ratio in IDR1b-18YS compared to the wild-type ARID1A IDR1b, from approximately 40% to less than 10% (Fig. 1G). These findings strongly suggest that tyrosine mutations disrupt the phase separation properties of IDR1b. Additionally, we applied protein intrinsic disorder prediction and structural composition analysis to identify the intrinsically disordered regions (IDRs) of two other subunits in the cBAF complexes, BRG1 and SS18 (Fig. S1F and S1G). However, the similar droplet formation assays and sedimentation assays indicated that these IDRs did not exhibit significant phase separation properties under these conditions (Figs. 1F, 1G and S1I). Collectively, our findings highlight that among the cBAF complexes, ARID1A IDR1b demonstrates the most pronounced liquid–liquid phase separation behavior.

ARID1A IDR1b, which is rich in SYGQ amino acids, exhibits the capability for protein liquid-liquid phase separation *in vitro*. This property strongly resembles the behavior of EWS-FLI1 IDR. Based on our TurboID experiments showing that EWS-FLI1 and ARID1A were spatially proximal within the cells (Fig. 1C), we speculate that EWS-FLI1 may interact with IDR1b directly through phase separation. To test this possibility, we conducted droplet colocalization experiments using EWS-FLI1 and several IDRs from cBAF subunits. The results revealed that IDR1b formed droplets clearly colocalized with the droplets formed by EWS-FLI1 (Fig. 1H). Additionally, both IDR1b and EWS-FLI1 mutually enhanced each other’s droplet formation (Fig. 1H). However, under identical experimental conditions, neither the IDR1b-18YS mutant nor IDRs from other cBAF subunits exhibited droplet formation driven by EWS-FLI1 (Fig. 1I). We noticed that in our *in vitro* droplet formation experiments, purified fusion proteins of EWS-FLI1 tended to aggregate irregularly over prolonged reaction times, as shown in Fig. 1I. However, the presence of IDR1b helped maintain the distinctive droplet structure of EWS-FLI1. It is important to note that protein phase separation processes are dynamic and reversible, and play a crucial role in their functionality. Therefore, the fact that IDR1b contributed to preserving the droplet morphology of EWS-FLI1 supports our conclusion of a direct interaction between IDR1b and phase-separated EWS-FLI1.

To determine whether the IDR1 is essential for the recruitment of the cBAF complex to genes targeted by EWS-FLI1 *in vivo*, we aimed to generate specific truncations of IDR1. The objective was to create mutants that would not inherently disrupt the chromatin remodeling activity of the cBAF complex. By doing so, we sought to ensure that any observed misregulation of target genes could be attributed directly to the inability of the mutant cBAF complex to interact with the phase-separated EWS-FLI1, rather than a loss of cBAF’s intrinsic remodeling capabilities. First, we employed an eukaryotic expression system to purify cBAF complexes containing wild-type ARID1A (WT), ARID1A ΔIDR1 and ARID1A ΔIDR1b mutants (Fig. S2A). Stable expression of Flag-tagged ARID1A WT, ΔIDR1, and ΔIDR1b was achieved in HEK293T cells using lentivirus infection. High-quality cBAF complexes were obtained by performing Flag immunoprecipitation (IP) from nuclear extract, followed by purification through glycerol gradient centrifugation (Figs. 2A, 2B and S2B). Deletion of IDR1 and IDR1b did not exhibit any noticeable effect on the overall integrity of the cBAF complexes.

**Figure 2.**
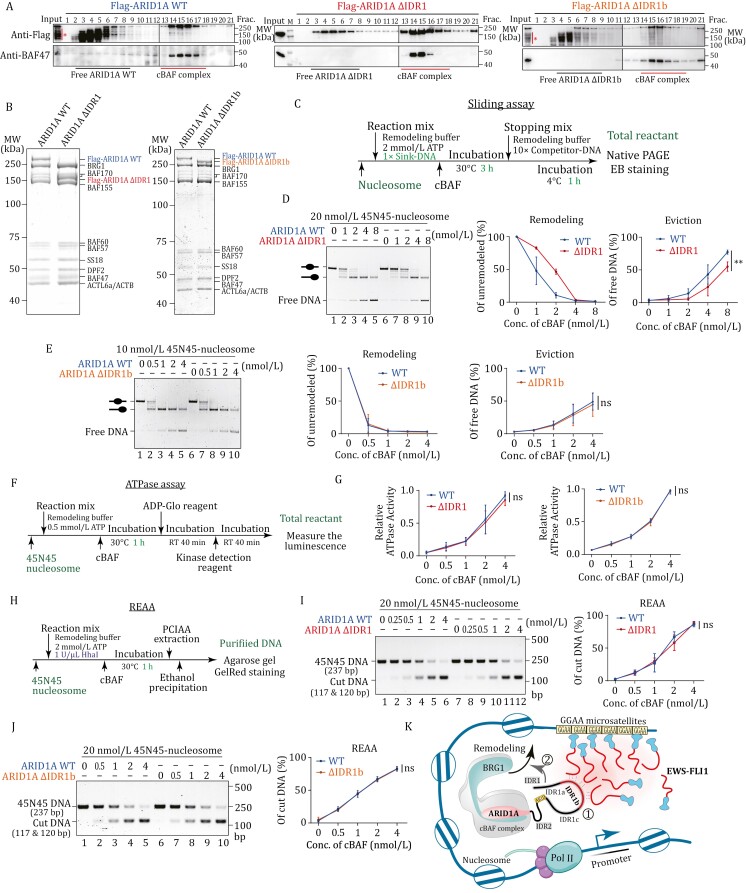
ARID1A IDR1 contributes to nucleosome remodeling activity of cBAF ***in vitro***. (A) Immunoblotting shows glycerol gradient sedimentation of Flag-purified cBAF complexes containing Flag-tagged ARID1A wild-type (right), ΔIDR1 (middle), or ΔIDR1b (left). The degradations of ARID1A WT or ΔIDR1b are indicated with stars. (B) Purified cBAF complexes containing Flag-tagged ARID1A wild-type, ΔIDR1, or ΔIDR1b are presented on SDS-PAGE and stained with Coomassie blue. (C) Workflow of the *in vitro* sliding assay. (D) Sliding assay and its quantification diagrams of 45N45-nucleosome and cBAF complexes containing ARID1A WT or ΔIDR1. The sliding reaction is loaded on native PAGE and stained with ethidium bromide. The remodeling diagram represents the amount of 45N45-nucleosome substrate, and the Eviction diagram represents the amount of free 45N45-DNA product. The data comprise three individual replicates and are represented as mean ± SD. The *P*-value is calculated by unpaired Student’s *t*-test. ** indicates *P*-value < 0.01; “ns” indicates not significant. (E) Sliding assay and its quantification diagrams of 45N45-nucleosome and cBAF complexes containing ARID1A WT or ΔIDR1b. (F) Workflow of the *in vitro* ATPase assay. (G) Quantification of ATPase assays with indicated concentrations of cBAF complexes. The data comprise three individual replicates and are represented as mean ± SD; “ns” indicates not significant. (H) Workflow of the *in vitro* restriction enzyme accessibility assay (REAA). (I) Restriction enzyme accessibility assay and its quantification diagram of cBAF complexes containing ARID1A WT or ΔIDR1. The DNA product in REAA is presented on agarose gel and stained with Gel-Red dye. The data comprise three individual replicates and are represented as mean ± SD; “ns” indicates not significant. (J) Restriction enzyme accessibility assay and its quantification diagram of cBAF complexes containing ARID1A WT or ΔIDR1b. The DNA product in REAA is presented on agarose gel and stained with Gel-Red dye. The data comprise three individual replicates and are represented as mean ± SD; “ns” indicates not significant. (K) Schematic of the mechanism by which ARID1A IDR targets EWS-FLI1 condensates and finetunes chromatin remodeling.

To establish our nucleosome sliding assays, we reconstituted center-positioned (45N45) and lateral-positioned (216L) nucleosomes using purified DNA and HeLa core histones. These assays, widely acknowledged as reliable techniques for evaluating nucleosome movement and remodeling activity (Huh et al., 2012), involved the addition of sink-DNA to absorb evicted histones from remodeled nucleosomes. Our nucleosome sliding experiments revealed that the absence of IDR1 significantly impairs the sliding activity of the mutant cBAF complex, as evidenced by a decrease in unremodeled nucleosome substrate and an increase in evicted free DNA product (Figs. 2C, 2D and S2C). To determine if ΔIDR1 directly affects the motor activity of BRG1 ATPase, we measured the ATPase activity of these cBAF complexes, and no significant differences were observed in the presence or absence of sink-DNA (Figs. 2F, 2G and S2E). Interestingly, ΔIDR1b did not appear to influence sliding activity or ATPase activity of cBAF complex comparing to ARID1A WT (Fig. 2E and 2G), suggesting that the regulatory roles of IDR1a and IDR1c may contribute to important regulatory roles in coordinating cBAF activities.

During the course of our study, we became aware of a recently published paper (Patil et al., 2023) in which Patil et al. reported that the complete removal of IDR1 does not affect the remodeling activity of cBAF via ATPase assay and Restriction Enzyme Accessibility Assay (REAA). To reconcile this apparent discrepancy, we performed a similar the Restriction Enzyme Accessibility Assay. In this assay, we employed the *Hha*I endonuclease to cleave the 237bp 45N45 nucleosomal DNA template into two fragments (120 bp and 117 bp) when the restriction site was accessible. Remarkably, our experimental findings revealed no substantial disparities in chromatin accessibility between the ARID1A WT and ΔIDR1 mutant cBAF (Fig. 2H–J). Likewise, the removal of IDR1b did not affect the pattern of accessibility changes during nucleosome remodeling by cBAF (Fig. 2J). These observations indicate that the observed inconsistency in the sliding assay cannot be ascribed to inaccurate quantification of the complexes or the specific activity of the purified enzymes employed in this particular *in vitro* assay. It was observed that Patil et al. utilized the 263 bp 50N66 nucleosome, which can be fragmented into 77 bp and 186 bp fragments by *Dpn*II endonuclease digestion. Hence, irrespective of the presence of restriction sites at the nucleosome DNA entry or the dyad axis, all these sites become accessible for cleavage during remodeling by WT and ΔIDR1 cBAF complexes. Based on these findings, we conclude that the presence of IDR1 does not significantly affect the ATPase activity of the cBAF complex or the initiation of nucleosome remodeling reactions. However, IDR1 does play a crucial role in histone eviction and histone octamer sliding along DNA.

Building on the finding that deletion of the IDR1b (ΔIDR1b) did not compromise the chromatin remodeling activity of the cBAF complex, we proceeded to evaluate the role of the ARID1A IDR1b in recruiting the cBAF complex to target genes in the context of the EWS-FLI1 fusion protein *in vivo*. To this end, we designed an overexpression study in an ARID1A-knockout HEK293T cell line. We introduced expression constructs for wild-type ARID1A (ARID1A WT) and ARID1A with the IDR1b deleted (ARID1A ΔIDR1b), along with the EWS-FLI1 fusion gene. Western blot analysis confirmed successful expression of ARID1A WT, ARID1A ΔIDR1b, and EWS-FLI1 in the knockout cells (Fig. S3A). Subsequent RNA sequencing (RNA-seq) analysis allowed us to compare gene expression profiles under different conditions. Specifically, we identified 3904 genes whose expression was altered by the presence of ARID1A WT in conjunction with EWS-FLI1 (ARID1A WT + EWS-FLI1 group), compared to cells expressing EWS-FLI1 with an empty vector (Empty + EWS-FLI1 group). Additionally, comparing the ARID1A WT + EWS-FLI1 group to the ARID1A WT + control group (Ctrl), we found that 797 genes were specifically influenced by EWS-FLI1 when ARID1A was present. The overlap between these two groups revealed 325 genes that were co-regulated by ARID1A and EWS-FLI1 (Fig. S3B). To discern the specific contributions of the ARID1A WT and ARID1A ΔIDR1b to the regulation of these co-regulated genes, we performed principal component analysis (PCA). The PCA revealed distinct clustering of the ARID1A ΔIDR1b samples, which differed from both the control and ARID1A WT, within the space of the ARID1A and EWS-FLI1 co-regulated genes. This pattern suggests that the IDR1b of ARID1A is critical for the regulation of gene transcription driven by EWS-FLI1 (Fig. S3C). Further analysis subdivided the ARID1A- and EWS-FLI1-dependent genes into four clusters. Notably, gene clusters 2 and 4 showed significant reliance on the IDR1b for their expression, underscoring the importance of IDR1b in controlling these specific sets of genes (Fig. S3D). In conclusion, the RNA-seq data provide strong evidence that the IDR1b of ARID1A is a key factor in the transcriptional regulation of genes targeted by EWS-FLI1, suggesting that IDR1b may contribute to the targeted recruitment of the cBAF complex by EWS-FLI1 to these genes.

Although there have been notable advancements in structural investigations of cBAF and PBAF complexes (He et al., 2020), numerous essential functional roles of IDR within various subunits are yet to be unveiled. Our study has revealed that the deletion of ARID1A IDR1 in the cBAF complex leads to a significant reduction in nucleosome sliding activities, while the ATPase activity of the complex remains unaffected. Interestingly, when comparing the results obtained from the restriction enzyme accessibility assays with the sliding assays, we found that the removal of IDR1 from ARID1A does not hinder the cBAF complex’s ability to enhance accessibility around the nucleosome dyad. It is worth noting that the sliding assay detects a greater number of remodeling intermediates, providing a more comprehensive understanding of the remodeling process. Importantly, we observed differences between the wild-type (WT) and ΔIDR1 samples, but not with the smaller ΔIDR1b, thereby reinforcing the reliability and robustness of our experimental system. This observation suggests potential mechanisms underlying the nucleosome remodeling process of the cBAF complex. These mechanisms can be categorized into two distinct processes: relaxation of nucleosomal DNA-histone interactions and sliding/displacement of the histone octamer along DNA templates. IDR1 may directly participate in the latter process or act as a bridge between the two processes. This proposed mechanism aligns with the loop/bulge propagation model of nucleosome sliding (Nodelman and Bowman, 2021). Alternatively, IDR1 might regulate the sliding direction of cBAF. In the absence of IDR1, cBAF could exhibit bidirectional movement on the nucleosome templates, resulting in observable bulges in the REAA. However, such bouncing motion could significantly reduce the efficiency of producing slide-away nucleosomes or histone eviction, detectable through our more sensitive sliding assay. Furthermore, based on the twist diffusion model, where ATPase-dependent twist diffusion induces rotation of the DNA duplex, exposing restriction sites (Nodelman and Bowman, 2021), IDR1 may not directly participate in DNA translocation but instead regulate the remodeling process by controlling the interaction between the remodeler and histones. To gain further insights and elucidate the underlying mechanisms, future studies could employ single-molecule-based biophysical approaches for detailed resolution.

Chromatin remodeling is primarily governed by the targeted recruitment of transcription factors and local retention, which is influenced by local chromatin characteristics (Hassan et al., 2001). The activity of chromatin remodeling is tightly regulated by intrinsic mechanisms exerted by distinct subunits, ensuring precise chromatin rearrangement (Clapier et al., 2017). These subunits contribute to ATPase-dependent DNA translocation, binding to the nucleosome acidic patch (Clapier et al., 2017), interacting with other regulators or histone modifications (Zhang et al., 2020), and internal stabilization (Mashtalir et al., 2018). Previous research extensively investigated the impact of subunit loss or mutation on tumor occurrence and development, revealing significant correlations between variations in human SWI/SNF subunits, genomic occupancy, and cellular functions (Clapier et al., 2017). However, the role of these subunits in external communication or internal regulation remains uncertain due to limited studies. In our study, we elucidated two distinct functions of the IDRs in ARID1A within the cBAF complex: facilitating transcription factor-dependent targeting and regulating the remodeling process (Fig. 2K). The proposed model suggests that ARID1A IDR1 specifically localizes to the phase-separated condensates formed by the EWS-FLI1 fusion protein. This targeted interaction facilitates the recruitment of cBAF to specific genes, leading to altered gene expression patterns. Additionally, the ARID1A IDR1 is posited to exert a regulatory influence on the inherent chromatin remodeling activities of cBAF. Intracellular proteins often possess abundant intrinsically disordered regions. Recent studies have extensively reported the occurrence of liquid-liquid phase separation in IDRs containing specific amino acids (Hyman et al., 2014). However, the limited specificity of protein phase separation poses challenges for comprehensive biological regulation. For instance, immunofluorescence analysis confirmed that p300 levels in the nucleus remained unchanged, despite the lack of colocalization between p300 and cBAF complexes carrying FUS/DDX4IDR-ARID1A fusions, indicating the inability of chimeric proteins to effectively interact with their correct binding partners (Patil et al., 2023). Our study demonstrates that the liquid-liquid phase separation of the ARID1A subunit of cBAF facilitates the interaction between the remodeler and fusion transcription factors, highlighting the recruitment of ARID1A by EWS-FLI1 through compatible multivalency-mediated phase separation. In conclusion, our study provides new insights into the multifaceted nature of ARID1A IDR1 and its impact on cBAF function, emphasizing the need for further exploration and a deeper understanding of the complex regulatory mechanisms involved. Given the significant role of ARID1A in cancers, future comprehensive investigations into the structure and function of ARID1A will be crucial for developing effective treatment strategies for relevant tumors.

## Supplementary information

The online version contains supplementary material available at https://doi.org/10.1093/procel/pwae029. The raw RNA-seq data from this study have been deposited in the GEO database under accession number GSE263234.

pwae029_suppl_Supplementary_Figures_S1-S3

pwae029_suppl_Supplementary_Table

## Footnotes

We thank the proteomics platform of Core Facility of Basic Medical Sciences, Shanghai Jiao Tong University School of Medicine and Shanghai Frontiers Science Center of Cellular Homeostasis and Human Diseases. We thank Dr. Dan Li at SJTU for technical assistance. This work was supported by the National Key R&D Program of China (2021YFA1300100 to B.L.), the National Natural Science Foundation of China (32030019, 31872817 to B.L., 82072892 to J.W., 81901284 to YH.Y., 32000413 to J.S., 31970587, 32170609 to X.S.), the Natural Science Foundation of Guangxi (No. 2023GXNSFAA026276 to J.W.) and the Fundamental Research Funds for the Central Universities. B.L. and J.X. designed the study with contributions of other authors; J.X. and J.W. performed remodeling assays, Y.P., N.L., and J.X. performed phase separation assays; S.L., J.W., and J.X. performed TurboID assay; M.Y., X.X., and J.X. prepared plasmids and proteins purification; Q.Z., Y.L., and J.X. performed MS analysis; F.L. and X.S. constructed cell lines; B.L. and J.X. wrote the manuscript; B.L. supervised the project. Authors declare no competing interests.
