# Supplementary material for: ARID1A IDR targets EWS-FLI1 condensates and finetunes chromatin remodeling
Source: Protein Cell. 2024 May 18;16(1):64–71. doi: 10.1093/procel/pwae029 (PMC11700579; doi:10.1093/procel/pwae029)
Supplement: pwae029_suppl_Supplementary_Figures_S1-S3 [file pwae029_suppl_supplementary_figures_s1-s3.pdf]

ARID1A IDR Targets EWS-FLI1 Condensates and Finetunes Chromatin Remodeling

Jingdong Xue[1], Siang Lv[2,3,4], Ming Yu[1], Yixuan Pan[1], Ningzhe Li[1,5], Xiang Xu[2], Qi Zhang[1], Mengyuan Peng[1], Fang Liu[1], Xuxu Sun[1], Yimin Lao[1], Yanhua Yao[1], Juan Song[1], Jun Wu[2,3,4]* and Bing Li[1]*

[1] Department of Biochemistry and Molecular Cell Biology, Key Laboratory of Cell Differentiation and Apoptosis of Chinese Ministry of Education, Shanghai Key Laboratory for Tumor Microenvironment and Inflammation, Shanghai Jiao Tong University School of Medicine, Shanghai, 200025, China.

[2] Department of Laboratory Medicine, Jiading Branch of Shanghai General Hospital, Shanghai Jiao Tong University School of Medicine, Shanghai, 201803, China.

[3] Clinicopathological Diagnosis & Research Center, The Affiliated Hospital of Youjiang Medical University for Nationalities, Baise, 533000, China.

[4] Key Laboratory of Tumor Molecular Pathology of Guangxi Higher Education Institutes, Baise, 533000, China.

[5] Shanghai Institute of Hematology, State Key Laboratory of Medical Genomics, National Research Center for Translational Medicine at Shanghai, Ruijin Hospital Affiliated to Shanghai Jiao Tong University School of Medicine, School of Life Sciences & Biotechnology, Shanghai Jiao Tong University, Shanghai, 200025, China.

* Correspondence: jun.wu@shsmu.edu.cn; bingli@shsmu.edu.cn

Supplementary information

MATERIAL AND METHODS

Plasmid construction

The cDNA of IDRs of ARID1A, SMARCA4, and SS18, as well as their variants, were cloned into the pRET-GST-mCherry bacterial expression vector using the 5'-XhoI and 3'-NotI restriction sites. The cDNA of the fusion protein EWS-FLI1 was cloned into the pRSF-His-EGFP bacterial expression vector using the 5'-XhoI and 3'-NotI restriction sites. The DNA fragment of TurboID was synthesized by GENEWIZE based on the DNA sequence from Addgene (Plasmid #107171) (Branon et al., 2018) . The TurboID fragment containing the 5'-BamHI and 3'-XhoI restriction sites, and EWS-FLI1 or EWS(YS)-FLI1 containing the 5'-XhoI and 3'-NotI restriction sites, were cleaved and cloned into the pCDH-CMV-Flag lentivirus expression vector containing the 5'-BamHI and 3'-NotI restriction sites. The cDNA of ARID1A ΔIDR1 and ΔIDR1b were constructed through overlapping PCR. The ARID1A WT and its truncations were cloned into the pCDH-CMV-Flag lentivirus expression vector using the 5'-XhoI and 3'-XbaI restriction sites.

TurboID assay

Flag-TurboID-EWS-FLI1 or EWS(YS)-FLI1 was stably expressed in HEK-293T cells through lentivirus infection. The plasmid mix (pMD2.G:pMDLg/pRRE:pRSV-Rev:pCDH = 3:5:2:5) was transfected into HEK-293T cells. The cells were cultured in a 10cm dish with DMEM supplemented with 10% FBS for 48 hours. The medium was collected as viral solution and filtered with a 0.45 µm filter. Fresh HEK-293T cells were infected with the filtered viral solution. After treatment with 1 µg/ml puromycin for 48 hours, western blot was performed to detect the expression of Flag-TurboID tagged EWS-FLI1 or EWS(YS)-FLI1.

The 293T cells expressing Flag-TurboID-EWS-FLI1 or EWS(YS)-FLI1 were plated in a 10 cm dish before labeling. The cells were cultured in complete DMEM medium containing 50 µM biotin for 6 hours at 37°C. After biotin treatment, the cells were washed with cold PBS and then digested with 0.5% trypsin. The cell pellet was washed with cold PBS twice and incubated in Hypotonic Buffer (10 mM Tris-HCl (pH 7.5), 1.5 mM $MgCl_2$, 10 mM KCl, 1 mM DTT, 1 mM PMSF) on ice for 10 minutes. The suspension was centrifuged at 3000 rpm at 4°C for 3 minutes, and the pellet was suspended in Hypotonic Buffer and homogenized using a KONTES Dounce homogenizer B-type pestle. The cell

nucleus was spun down at 10000 rpm at 4°C for 5 minutes and then resuspended with High-Salt Buffer (50 mM Tris-HCl (pH 7.5), 1 mM $MgCl_2$, 300 mM KCl, 1 mM EDTA, 1% NP-40, 1 mM DTT, 1 mM PMSF). The nucleus was incubated on a rotator at 4°C for 1 hour. The sample was centrifuged at 13000 rpm for 30 minutes at 4°C. The supernatant was collected as nuclear extract and quantified with Bradford before storing at -80°C.

The nuclear extract was thawed on ice and then incubated with pre-equilibrated Dynabead M-280 Streptavidin on a rotator at 4°C for 2 hours. The beads were then sequentially washed with High-Salt Buffer, 0.1 M Na_2CO_3, Tris-Urea Buffer (10 mM Tris-HCl (pH 8.0), 2 M Urea), and High-Salt Buffer. The beads were settled by putting the tube on a magnetic rack and the supernatant was removed carefully with a loading tip. The sample was eluted by adding SDS-loading buffer (60 mM Tris-HCl (pH 8.0), 2% SDS, 4 mM EDTA, 5 mM βME, 0.02% bromophenol blue, 10% glycerol) and incubating at 98°C for 10 minutes. The eluted protein was analyzed with western blot and mass spectrometry.

The mass spectrometry results were analyzed using the edgeR package in RStudio software. A volcano plot was generated to compare the spectral values of each protein between the EWS-FLI1 and EWS(YS)-FLI1 treatment groups. Proteins showing significant differences (Fold Change greater than 2 or less than 0.5, p-value less than 0.05) between these two treatment groups were subjected to Gene Ontology (GO) functional clustering analysis.

Western blot

The protein sample from the nuclear extract was mixed with 5× SDS-Loading Buffer (300 mM Tris-HCl (pH 8.0), 10% SDS, 20 mM EDTA, 25 mM βME, 0.1% bromophenol blue, 50% glycerol) at a 4:1 ratio and then incubated at 98°C for 10 minutes. The sample was separated on an 8% or 10% SDS-PAGE gel and transferred onto an NC membrane. The membrane was blocked with 10% milk in TBST at room temperature for 30 minutes and incubated with the primary antibody in 2% milk at 4°C overnight or at room temperature for 2 hours. After washing twice with 2% milk, the membrane was incubated with the secondary antibody in 2% milk at room temperature for 45 minutes. The membrane was sequentially washed twice with 2% milk and three times with TBST. The signal was detected with the Bio-Rad chemi channel after the addition of the ECL solution.

Protein purification

For the purification of GST-tagged BAF IDRs, the plasmids were transformed into E. coli

BL21(DE3) pLysS. Multiple colonies were picked from freshly transformed plates and inoculated into LB + Ampicillin (200 μg/mL) media at 37°C. Once the OD_{600} reached 0.4, the culture was chilled to room temperature. Protein overexpression was induced by adding 0.2 mM IPTG. The culture was vigorously shaken at 16°C overnight. Cells were washed with cold STE Buffer (10 mM Tris-HCl (pH 8.0), 100 mM NaCl, 1 mM EDTA) and resuspended in GST-2M Buffer (2 M NaCl, 10 mM Tris-HCl (pH 7.5), 20 mM Na_3PO_4 (pH 6.8), 10 mM βME, 0.01% NP-40). The suspension was sonicated 3 times at 30% power and a duty cycle of 90% for 15 seconds at 4°C. 1% Triton X-100 was added, and the lysate was sonicated once again. Insoluble fractions were removed by centrifugation at 12,000 rpm for 20 minutes at 4°C. The lysate was incubated with pre-equilibrated Glutathione Sepharose beads for 4 hours at 4°C. The beads were sequentially washed with 10 column volumes of GST-1 M Buffer (1 M NaCl, 10 mM Tris-HCl (pH 7.5), 20 mM Na_3PO_4 (pH 6.8), 10 mM βME, 0.01% NP-40) and GST-0.1 M Buffer (0.1 M NaCl, 10 mM Tris-HCl (pH 7.5), 20 mM Na_3PO_4 (pH 6.8), 10 mM βME, 0.01% NP-40). The protein was eluted with 300 mM glutathione dissolved in GST-Elution Buffer (25 mM Tris (pH 8.0), 50 mM NaCl, 10 mM BME, 10% glycerol). The eluted protein was dialyzed with GST-Elution Buffer overnight and then concentrated using a Millipore 10 KD ultrafilter.

The purification of His-EGFP-tagged EWS-FLI1 was previously described (Zuo et al., 2021). The culture of bacteria and induction of protein expression were the same as described above for GST purification. The bacteria were suspended and sonicated in His-Lysis Buffer (50 mM Tris-HCl (pH 7.4), 1 M KCl, 1 M Urea, 10 mM Imidazole, 1.5 mM BME, 5% glycerol). The supernatant was collected after centrifugation at 12,000 rpm for 20 minutes at 4°C. The lysate was incubated with pre-equilibrated Ni-NTA beads for 2 hours at 4°C. The beads were washed with His-Wash Buffer (50 mM Tris-HCl (pH 7.4), 1 M KCl, 1 M Urea, 50 mM Imidazole, 1.5 mM βME, 5% glycerol). The His-tagged protein was eluted with His-Elution Buffer (50 mM Tris-HCl (pH 7.4), 1 M KCl, 1 M Urea, 500 mM Imidazole, 1.5 mM βME, 5% glycerol) and then further purified with a GE Superdex 200 column in His-Lysis Buffer. The fractions of protein were selected and combined according to the UV260 and UV280 curves. The protein was concentrated using a Millipore 10 KD ultrafilter and stored at -80°C.

Glycerol gradient sedimentation assay

A linear gradient of 10-30% glycerol was prepared in a 10 mL centrifuge tube (Beckman Coulter). 5 mL of Flag-Elution Buffer (30% glycerol) was added to the bottom of the centrifuge tube, and 5 mL of Flag-Elution Buffer (10% glycerol) was slowly and gently added to the centrifuge tube along the wall.

The lid was closed, and the centrifuge tube was gently and gradually tilted. After holding at 4°C for 4 hours, the centrifuge tube was slowly uprighted. The protein sample was gently loaded along the tube wall to the liquid surface in the centrifuge tube. The samples were centrifuged in a Beckmann ultracentrifuge SW40 rotor at 40,000 rpm at 4°C for 16 hours. After centrifugation, the samples were removed by 0.5 mL per fraction and analyzed with immunoblotting.

BAF complex purification

For the purification of BAF complexes containing Flag-tagged wild-type and variant ARID1A, the plasmid mix (pMD2.G: pMDLg/pRRE: pRSV-Rev: pCDH = 3: 5: 2: 5) was transfected into HEK-293T cells. The cells were cultured in a 10cm dish with DMEM supplemented with 10% FBS for 48 hours. The medium was collected and filtered with a 0.45 μm filter. Fresh HEK-293T cells were infected with the filtered viral solution. After treatment with puromycin for 48 hours, the cells were digested using 0.5% trypsin, washed once with PBS, and transferred to a glass shaker for extended culture at 37°C, 5% CO_2, and 170 rpm in serum-free medium.

Once the cell density reached 8×10^6 cells/mL, they were collected by centrifugation at 3500 rpm for 5 minutes at 4°C. The cells were washed once with PBS and then suspended in a Hypotonic Buffer (10 mM Tris-HCl (pH 7.5), 10 mM KCl, 1.5 mM $MgCl_2$, 1 mM DTT, 1 mM PMSF) and incubated on ice for 10 minutes. The cells were spun down at 3000 rpm for 10 minutes at 4°C and resuspended in High-Salt Buffer (50 mM Tris-HCl (pH 7.5), 1 mM $MgCl_2$, 300 mM KCl, 1 mM EDTA, 1% NP-40, 1 mM DTT, 1 mM PMSF) and incubated at 4°C for 2 hours. Then, centrifuge at 13300 rpm for 60 minutes and collect the supernatant as cell extract.

The equilibrated Flag-beads were added to the cell extract and incubated at 4°C for 4 hours. The beads were spun down at 1200 rpm for 5 minutes at 4°C and sequentially washed with High-Salt Buffer and Flag-Elution Buffer (50 mM HEPES (pH 7.9), 100 mM NaCl, 2 mM $MgCl_2$, 0.02% NP-40; 10% Glycerol, 1 mM PMSF, 1 mM Benzamidine). The protein was eluted by 0.5 mg/mL 3×Flag peptide in Flag-Elution Buffer. The eluted protein was concentrated with a 30 KD ultrafilter to a volume of 200 μL and then separated with a 10%-30% glycerol gradient sedimentation assay. After centrifugation, samples were removed at 0.5 mL per fraction and then analyzed with western blot. The fractions containing BAF complex were combined according to the results of anti-Flag and anti-BAF47 in Western blot. The BAF complexes were concentrated with a 30 KD ultrafilter and stored at -80°C.

In vitro droplet assay

In vitro droplet formation was performed in a 96-well microplate. The droplet buffer and protein mix were prepared before the reaction. The default droplet buffer consisted of 50 mM Tris-HCl (pH 7.5), 50 mM NaCl, 5% glycerol, 8% PEG-8000, and 1 mM PMSF. In the droplet assay with a gradient of NaCl concentration, the droplet buffer contained twice the final concentration of NaCl. In the protein mix, the indicated protein was diluted to twice the final concentration with GST-Elution Buffer (25 mM Tris-HCl (pH 8.0), 50 mM NaCl, 10 mM βME, 10% glycerol). An equal volume of droplet buffer and protein mix was sequentially added to the 96-well microplate, and the reaction was gently mixed through pipetting several times. The microplate was sealed with a polymethyl methacrylate membrane and incubated at room temperature for 60 minutes. The droplet formation was observed with a Nikon fluorescence microscope and phase contrast microscope. The quantification of the droplet assay was carried out with OpenCFU software.

Sedimentation assay

The sedimentation assay of the protein was performed in 0.6-mL tubes. Proteins were diluted to the indicated concentrations in a 10 μL Sedimentation Buffer (50 mM Tris-HCl (pH 7.5), 50 mM NaCl, 5% glycerol, 1 mM PMSF, 8% PEG-8000). The mixtures were gently mixed through pipetting several times. The mixed samples were incubated at 18°C for 30 minutes and then centrifuged at 12000 rpm for 1 minute at room temperature. The supernatant fractions were discarded, and the pellet fractions were analyzed with SDS-PAGE. The quantification of the sedimentation assay was carried out with ImageJ software.

Nucleosome construction

The 45N45-DNA was purified from the PCR product using plasmid #pBL-386 as the template. The primer pair #6300 and #6301 was used for the PCR, which was performed with Taq DNA polymerase (NEB). The resulting 237 bp PCR product was purified using the Bio-Rad 491 DNA purification system. The 216L-DNA was purified from the EcoRV (NEB) digested plasmid #pBL645, and the resulting 216 bp fragments were purified using the Bio-Rad 491 DNA purification system.

The purified core histones from HeLa, which were from the Li Lab stock, were used for reconstituting mono-nucleosomes with the purified DNA. The reconstitution was performed by step dilution. A 1 mL reaction containing core histones and DNA was mixed at a 1:1.2 ratio in TEB Buffer

(20 mM Tris-HCl (pH 7.5), 1 mM EDTA, 1 mM βME) supplemented with 2 M NaCl and incubated at 30°C for 2 hours. The reaction was then sequentially transferred to buffers supplemented with 1.2 M, 1 M, 0.8 M, and 0.6 M NaCl, and incubated at 30°C for 2 hours in each buffer. Finally, the reaction was transferred to TEB Buffer and incubated at 4°C for 2 hours.

The mono-nucleosome in the reaction was purified using the Bio-Rad 491 purification system conjugated with 3.5% native PAGE. The electrophoresis was carried out in 0.3×TBE Buffer at 20 W for 120 minutes at 4°C. The collection solution in the system was 10 mM Tris-HCl (pH 7.5). The fractions were analyzed with 3.5% native PAGE and ethidium bromide staining. The fractions containing mono-nucleosomes were combined and concentrated with a 30 KD ultrafilter. The nucleosomes were mixed with Stocking Buffer (250 mM NaCl, 5 mM βME, 50% Glycerol) at a 4:1 ratio. The nucleosomes were quickly frozen and stored at -80°C.

Sliding assay

The sliding assay was conducted following our established protocol with minor modifications, as described previously (Li et al., 2005; Carey et al., 2006; Huh et al., 2012). The purified BAF complexes were thawed on ice and then diluted with Flag-Elution buffer to a 15-fold indicated final concentration. The reaction mix was prepared on ice and consisted of Remodeling Buffer (20 mM Tris-HCl (pH 7.6), 50 mM KCl, 5 mM $MgCl_2$, 0.1 mg/mL BSA, 10% Glycerol, 1 mM PMSF) supplemented with the indicated nucleosome, 2 mM ATP, and 3 ng/μL sink-DNA (plasmid mix). The default stopping mix contained Remodeling Buffer supplemented with 225 ng/μL competitor-DNA (plasmid mix). To initiate the reaction, 1 μL of the BAF complex was added to 12 μL of the reaction mix. The reaction was thoroughly mixed and incubated at 30°C for 3 hours. Following incubation, 2 μL of the stopping mix was added and mixed into the reaction. The 15 μL reactant was incubated at 4°C for 1 hour and then loaded onto a 3.5% native polyacrylamide gel. Electrophoresis was carried out in 0.3×TBE buffer at 250 V for 150 minutes at 4°C. The gel was stained with 50 μg/mL ethidium bromide for 1 hour and destained with water at room temperature. The DNA signal was detected with the Bio-Rad Ethidium bromide channel. The signal of the nucleosome substrate and free DNA product was quantified with ImageJ software.

ATPase assay

The purified BAF complexes were diluted with Flag-Elution buffer to a 15-fold indicated final concentration. The reaction mix was prepared on ice and consisted of Remodeling Buffer (20 mM Tris-HCl (pH 7.6), 50 mM KCl, 5 mM $MgCl_2$, 0.1 mg/mL BSA, 10% Glycerol, 1 mM PMSF) supplemented with 20 nM 45N45 nucleosome, 0.5 mM ATP, and the indicated concentration of sink-DNA. To initiate the reaction, 1 µL of the BAF complex was added to 14 µL of the reaction mix. The reaction was mixed thoroughly and incubated at 30°C for 1 hour or 3 hours. After incubation, the amount of ADP product in the reaction was measured using the ADP-Glo Kinase Kit (Promega). The luminescence signal was detected using the Molecular Device iD5 Luminescence channel.

Restriction enzyme accessibility assay

We performed the restriction enzyme accessibility assay following our previously established protocol, with slight adjustments as described below (Prochasson et al., 2003). The purified BAF complexes were diluted with Flag-Elution buffer to 15-fold of indicated final concentration. The Reaction mix was prepared on ice and consisted of Remodeling Buffer (20 mM Tris-HCl (pH 7.6), 50 mM KCl, 5 mM $MgCl_2$, 0.1 mg/mL BSA, 10% Glycerol, 1 mM PMSF) supplemented with 20 nM 45N45 nucleosome and 2 mM ATP. To initiate the reaction, 1 µL of the BAF complex was added into 14 µL of the Reaction mix. The reaction was mixed thoroughly and incubated at 30°C for 1 hour. After incubation, 45 µL water supplementary with 5 mM EDTA and 1 ng/µL control-DNA was added into the tube. The 60 µL reactant was mixed thoroughly with 60 µL PCIAA (Phenol: Chloroform: isoamyl alcohol = 25: 24: 1) and centrifuged at 12000 rpm for 15 minutes at 4°C. DNA in the supernatant was purified with ethanol precipitation and then analyzed with 1.5% agarose gel containing GelRed dye. The electrophoresis was carried out in 1× TAE buffer at 180 V for 30 minutes. The signal was detected with Bio-Rad GelRed channel.

RNA-seq analysis

The cell culture medium in the culture plate was removed, and the cells were washed with pre-cooled DEPC-treated PBS. Then, 1 mL of Trizol reagent was added to lyse the cells. The cell lysate was transferred to a 1.5 mL RNase-free centrifuge tube. Next, 200 µL of chloroform was added to the tube, followed by centrifugation. The RNA in the aqueous phase was isolated through isopropanol precipitation and subsequently washed with 75% ethanol. The RNA was dissolved in RNase-free water for further analysis. The sequencing libraries were constructed using 1 µg of the total RNA with the

Abclonal Fast RNA-seq Lib Prep Kit. Sequencing was performed by Novogene on an Illumina NovaSeq 6000 instrument. The filtered RNA-seq data were analyzed using MobaXterm software. Alignment of the reads to the reference genome GRCh38 (hg38) was performed using the STAR tool. PCR duplicates were removed from the mapped reads using the Samtools 'rmdup' tool. Gene expression data for each group were calculated using the FeatureCounts tool. The gene expression results were analyzed using the edgeR package in RStudio software.

Immunofluorescence Staining

The freshly cultured cell suspension was added to a 12-well plate, with a sterile glass slide placed in each well. The cells were maintained in DMEM supplemented with 10% fetal bovine serum (FBS) and antibiotics in a humidified atmosphere with 5% CO_2 at 37°C. When immunofluorescence procedures were initiated, the medium was removed, and the cells were washed once with 1 mL PBS. Then, 0.5 mL of 4% paraformaldehyde was added to the cells and incubated at room temperature for 15 minutes to fix the cells. Subsequently, the cells were permeabilized with 0.5 mL of 0.3% Triton X-100 for 5 minutes at room temperature. The cells were then blocked with 0.5 mL of 1% BSA for 30 minutes at room temperature. The cells were incubated overnight with the primary antibody diluted in 0.5% BSA at 4°C. Afterward, the cells were stained with an Alexa Fluor 546-conjugated secondary antibody for 2 hours at room temperature. The slides were mounted with ProLong Gold Antifade Mountant with DAPI. The samples were observed and photographed using a Nikon fluorescence microscope.

Supplemental Table 1. The list of plasmids used in this study

Plasmids	Backbone	Parental	Plasmid description	Source
#pMD001	pRET		pRET-GST-EGFP-Flag	this study
#pMD002	pRET		pRET-GST-mCherry-Flag	this study
#pMD018	pRSF		pRSF-EGFP-EWS-FLI1	this study
#pMD041			pMD2.G	Li Lab
#pMD044			pMDLg/pRRE	Li Lab
#pMD045			pRSV-Rev	Li Lab
#pMD106	pRET	#pMD002	pRET-GST-SS18 IDR2mCherry-2xFLAG	this study
#pMD107	pRET	#pMD002	pRET-GST-SS18 IDR1-mCherry-2xFLAG	this study
#pMD108	pRET	#pMD002	pRET-GST-BRG1 IDR1-mCherry-2xFLAG	this study
#pMD109	pRET	#pMD002	pRET-GST-BRG1 IDR2-mCherry-2xFLAG	this study
#pMD112	pRET	#pMD002	pRET-GST-ARID1A IDR1a mCherry-2xFLAG	this study
#pMD129	pcDNA3.1		pcDNA3.1-Flag-EGFP-dna	this study
#pMD134	pcDNA3.1	#pMD129	pcDNA3.1-Flag-EGFP-ARID1A N	this study
#pMD135	pcDNA3.1	#pMD129	pcDNA3.1-Flag-EGFP-SS18 N	this study
#pMD136	pcDNA3.1	#pMD129	pcDNA3.1-Flag-EGFP-SS18 C	this study
#pMD137	pcDNA3.1	#pMD129	pcDNA3.1-Flag-EGFP-SS18	this study
#pMD141	pcDNA3.1	#pMD129	pcDNA3.1-Flag-EGFP-ARID1A C	this study
#pMD191	pCDH		pCDH-CMV-Flag-ARID1A	this study
#pMD195	pCDH		pCDH-CMV-Flag-ARID1A(IDR-del)	this study
#pMD312			12x216L DNA (pBL-645)	Li Lab
#pMD350	pGEM		45N45 nucleosomal DNA (pBL-386)	Li Lab
#pMD351	pCDH		pCDH-Flag-TurboID-NLS	this study
#pMD353	pCDH		pCDH-Flag-TurboID-EWS-FLI1	this study
#pMD354	pCDH		pCDH-Flag-TurboID-EWS(YS)-FLI1	this study
#pMD371	pRET	#pMD002	pRET-GST-ARID1A IDR1a(18YS)-mCherry	this study
#pMD372	pCDH		pCDH-CMV-Flag-ARID1A(IDR1b-del)	this study
#pMD373	pcDNA3.1		pcDNA3.1-EGPF	this study
#pMD374	pcDNA3.1	#pMD373	pcDNA3.1-EGPF-EWS-FLI1	this study

Supplemental Table 2. The list of primers used in this study

Primers	Name	Sequence
2353	EWSR1 1F	tccctcgagatggcgtccacggattacag
2427	SS18 1F XhoII	tccctcgagATGTCTGTGGCTTTCGCGG
2428	SS18 1257R stop NotI	catgcggccgcTCACTGCTGGTAATTTCCAT
2566	FLI1 stop NotI R	catgcggccgcTTAGTAGTAGCTGCCTAAG
2755	SS18-IDR-F	TCACTCGAGGATTCTAATCAAAATATGCAG
2756	SS18-IDR-R	GACGCGGCCGCTGAGTACTGCTGTGGTGGGCC
2761	BRG1-IDR1-F	ATCCTCGAGatgtccactccagaccca
2762	BRG1-IDR1-R	CTGGCGGCCGCgttctgctgccccaagg
2763	BRG1-IDR2-F	TACCTCGAGcccctccccgaccacctgca
2764	BRG1-IDR2-R	CTCGCGGCCGCctcctgcaggatctccacagggt
2994	ARID1A IDR F	atcctcgagtcgggaggggcccaacaaag
2995	ARID1A IDR R	ATCGCGGCCGCgctggctgccatccctgcag
3681	SS18 IDR2 F	accctcgagGGCCAACAGCAAGATGCAT
3701	ARID1A 1026R Not1	atcctcgagcgccgccgcagccccccaac
4035	ARID1A 2893F XhoI+AgeI	ATCCTCGAGACCGGTccagagatgatgggccttgg
4036	ARID1A 2892R XbaI+AgeI	ATCTCTAGAACCGGTgctggctgccatccctgcag
4038	Arid1A-Not1-TAA-R	ctagcggccgcTCATGACTGGCCAATCAAAAACAGTACATCA
6300	BL_45N45_F	AACACTATCCGACTGGCACCGGC
6301	BL_45N45_R	ACACCGAGTTCATCCCTTATGTGATG
6565	TurboID_BamHI_F	ATGCGGATCCATGAAAGACAATACTGTGCCTCTGAAG
6569	Turbo_XhoI_R	ATGCTCGAGGCTAGCCTTTTCGGCAGACCG
6570	XhoI_NLS_NotI_F	tcgagCCAGCAGCGAAGAAAAAGAAGCTGGATtaggc
6571	XhoI_NLS_NotI_R	GGCCgcctaATCCAGCTTCTTTTTCTTCGCTGCTGGc
MDprimer-815	IDR-dele2-F	cggcagctgcggcggcggccgccgagctatctcaagattcatttg
MDprimer-816	IDR-dele2-R	ttgagatagctcggcggccgccgccgcagctgccgccgccgcagcccc
MDprimer-817	HindIII_EGFP_F	GTTTAAACTTAAGCTTATGGTGAGCAAGGGCGAGGAG
MDprimer-818	XhoI_EGFP_R	ATCGATCTCGAGGGATCCCTTGTACAGCTCGTCCATGCC

Supplemental Table 3. The list of reagents or resource used in this study

REAGENT or RESOURCE	SOURCE	IDENTIFIER
**Antibodies**		
SMARCB1/BAF47 Rabbit mAb	CST	D8M1X
Flag-HRP	Sigma	A8592
Peroxidase AffiniPure Goat Anti-Mouse IgG (H+L)	AffiniPure	115-035-003
Peroxidase AffiniPure Goat Anti-Rabbit IgG (H+L)	AffiniPure	111-035-003
BAF250 Polyclonal Antibody	Abclonal	A16648
FLAG M2 mouse monoclonal antibody	sigma	F1804
Goat anti-Mouse IgG (H+L) Alexa Fluor 546	Invitrogen	A11003
Rabbit Anti-FLI1 antibody	Abcam	ab15289
ACTB Monoclonal Antibody (mouse)	Abclonal	AC004
**Bacterial and virus strains**		
TOP10	Li Lab	N/A
BL21 DE3 pLysis codon+	Li Lab	N/A
**Chemicals, peptides, and recombinant proteins**		
Polyethylene Glycol 8000 (PEG)	Fisher	BP233
Dextran-70	BBI	A600375
Ficoll PM 400	Sigma	GE17-0300-50
Lipofectamine 3000 Transfection Reagent	Invitrogen	CW3007M
Puromycin Dihydrochloride	Gibco	A1113803
Dynabeads M-280 Streptavidin	Invitrogen	11205D
ATP solution	Sigma	GE27-2056-01
HhaI	NEB	R0139S
ProLong Gold Antifade Mountant with DAPI	Invitrogen	P36931
TRIzol Reagent	Invitrogen	15596018CN
Fast RNA-seq Lib Prep Kit V2	Abclonal	RK20306
GST-mCherry	This study	#pMD002
His-EGFP-EWS-FLI1	This study	#pMD018
GST-ARID1A IDR1b(WT)-mCherry	This study	#pMD112
GST-ARID1A IDR1b(18YS)-mCherry	This study	#pMD371
GST-BRG1 IDR1-mCherry	This study	#pMD108
GST-BRG1 IDR2-mCherry	This study	#pMD109
GST-SS18 IDR1-mCherry	This study	#pMD107
GST-SS18 IDR2-mCherry	This study	#pMD106
BAF complex (Flag-ARID1A WT)	This study	#pMD017
BAF complex (Flag-ARID1A WT) batch#2	This study	#pMD191
BAF complex (Flag-ARID1A ΔIDR1)	This study	#pMD195
BAF complex (Flag-ARID1A ΔIDR1b)	This study	#pMD372

Experimental models: Cell lines		
Human: HEK-293T	Li Lab	N/A
Human: ARID1A-knockout HEK-293T	Xuxu Sun	#12KO
Human: HeLa	Li Lab	N/A

Software and algorithms		
Fiji	Image J	https://imagej.net/imagej-wiki-static/Fiji
Photoshop	Adobe	https://www.adobe.com/cn/products/photoshop.html
Lightroom	Adobe	https://www.adobe.com/cn/products/photoshop-lightroom-classic.html
Image Lab	Bio-Rad	https://www.bio-rad.com/zh-cn/product/image-lab-software
OpenCFU	Quentin Geissmann	https://opencfu.sourceforge.net/
MobaXterm	mobatek	https://mobaxterm.mobatek.net/
STAR v2.7.10a	STAR	https://github.com/alexdobin/STAR
samtools v1.16.1	samtools	https://github.com/samtools/samtools
subread v2.0.1	subread	https://github.com/ShiLab-Bioinformatics/subread
RStudio	Posit	https://posit.co/downloads/
edgeR v3.18	Bioconductor	https://bioconductor.org/packages/release/bioc/html/edgeR.html

References:

Branon, T.C., Bosch, J.A., Sanchez, A.D., Udeshi, N.D., Svinkina, T., Carr, S.A., Feldman, J.L., Perrimon, N., and Ting, A.Y. (2018). Efficient proximity labeling in living cells and organisms with TurboID. *Nat Biotechnol* 2018; **36**: 880-887.

Carey, M., Li, B., and Workman, J.L. (2006). RSC Exploits Histone Acetylation to Abrogate the Nucleosomal Block to RNA Polymerase II Elongation. *Mol Cell* 2006; **24**: 481-487.

Huh, J.W., Wu, J., Lee, C.H., Yun, M., Gilada, D., Brautigam, C.A., and Li, B. (2012). Multivalent di-nucleosome recognition enables the Rpd3S histone deacetylase complex to tolerate decreased H3K36 methylation levels. *The EMBO journal* 2012; **31**: 3564-3574.

Li, B., Pattenden, S.G., Lee, D., Gutierrez, J., Chen, J., Seidel, C., Gerton, J., and Workman, J.L. (2005). Preferential occupancy of histone variant H2AZ at inactive promoters influences local histone modifications and chromatin remodeling. *Proc Natl Acad Sci U S A* 2005; **102**: 18385-18390.

Prochasson, P., Neely, K.E., Hassan, A.H., Li, B., and Workman, J.L. (2003). Targeting activity is required for SWI/SNF function in vivo and is accomplished through two partially redundant activator-interaction domains. *Mol Cell* 2003; **12**: 983-990.

Zuo, L., Zhang, G., Massett, M., Cheng, J., Guo, Z., Wang, L., Gao, Y., Li, R., Huang, X., Li, P., *et al.* (2021). Loci-specific phase separation of FET fusion oncoproteins promotes gene transcription. *Nature Communications* 2021; **12**.

Supplemental Figure Legends:

Figure S1 Phase-separated EWS-FLI1 recruits cBAF complex via ARID1A IDR1b

(a) Immunoblotting shows stable expression of Flag-tagged TurboID-EWS-FLI1 or TurboID-EWS(YS)-FLI1 in HEK-293T cells used in the TurboID assay.
(b) Immunoblotting shows that a number of nuclear proteins are labeled with biotin and enriched from 293T nuclear extract.
(c) GO analysis of the lost proteins (left) and gained proteins (right) in the TurboID assay. BP: Biological Process; CC: Cell Component; MF: Molecular Function.
(d) Structure information (top), predicted disorder score (middle), and amino acid composition analysis (bottom) of ARID1A. This is a supplement for Fig.1d.
(e) GST-ARID1A IDR1b-mCherry and its 18YS mutant on SDS-PAGE stained with Coomassie blue.
(f) Structure information (top), predicted disorder score (middle), and amino acid composition analysis (bottom) of BRG1.
(g) Structure information (top), predicted disorder score (middle), and amino acid composition analysis (bottom) of SS18.
(h) Representative images of mCherry-ARID1A IDR1b droplet formation in the presence of indicated reagents. The concentration of ARID1A IDR1b is 4 μM. Scale bar, 10 μm.
(i) Sedimentation assay and its quantification diagram for the SS18 IDRs. The sedimentation assay is resolved on SDS-PAGE and stained with Coomassie blue. The final concentration of each protein is 4 μM. The quantification data are representative of three individual repeats and are expressed as mean ± SD. ns indicates not significant; * indicates p-value < 0.05, as determined by an unpaired Student's t-test.
(j) Representative images of condensate formation in HeLa cells expressing indicated EGFP-tagged subunit fragments of the cBAF complex. The fragments are annotated in Fig.1d and Fig.S1g. Images in white boxes are magnified for detail and shown at the bottom as "zoom in". Scale bar, 10 μm.

Figure S2 ARID1A IDR1 contributes to nucleosome remodeling activity of cBAF complex in vitro

(a) Working flow of the purification of cBAF complexes from HEK-293T nuclear extract.
(b) Immunoblotting shows the glycerol gradient sedimentation of the second batch of Flag-purified cBAF complexes containing Flag-tagged ARID1A wild-type. The degradations of ARID1A WT are indicated with a red star.
(c) Sliding assay and its quantification diagrams of 216L-nucleosome and cBAF complexes containing ARID1A WT or ΔIDR1. The Remodeling diagram represents the amount of 216L-nucleosome substrate, and the Eviction diagram represents the amount of free 216L-DNA product. The data comprise three individual replicates and are represented as mean ± SD. The p-value is calculated by an unpaired Student's t-test. * indicates p-value < 0.05.
(d) Working flow of the in vitro ATPase activity assay with optimized conditions of additional Sink-DNA and reaction time.
(e) Quantification of ATPase assays for indicated concentrations of cBAF complexes containing ARID1A WT or ΔIDR1. The reaction is performed under the condition of Sink-DNA and 3 hours of incubation time. The p-value is calculated by an unpaired Student's t-test. ns indicates not significant.

Figure S3 ARID1A IDR1b plays an important role in the regulation of EWS-FLI1-dependent gene transcription

(a) Immunoblotting demonstrates the expression of ARID1A WT or ΔIDR1b and EWS-FLI1 in the ARID1A-knockout HEK-293T cell line.
(b) A Venn diagram illustrates the overlap between ARID1A-regulated genes and EWS-FLI1-regulated genes (Fold Change > 2 or < 0.5, p-value < 0.05) as revealed by RNA-seq analysis.
(c) A Principal Component Analysis (PCA) plot indicates that ΔIDR1b clusters distinctly from Ctrl or ARID1A WT among the genes co-regulated by EWS-FLI1 and ARID1A.
(d) A heatmap displays the relative RNA-seq gene expression levels of genes co-regulated by EWS-FLI1 and ARID1A in 293T cells (rows, n = 325 genes).

Figure S1

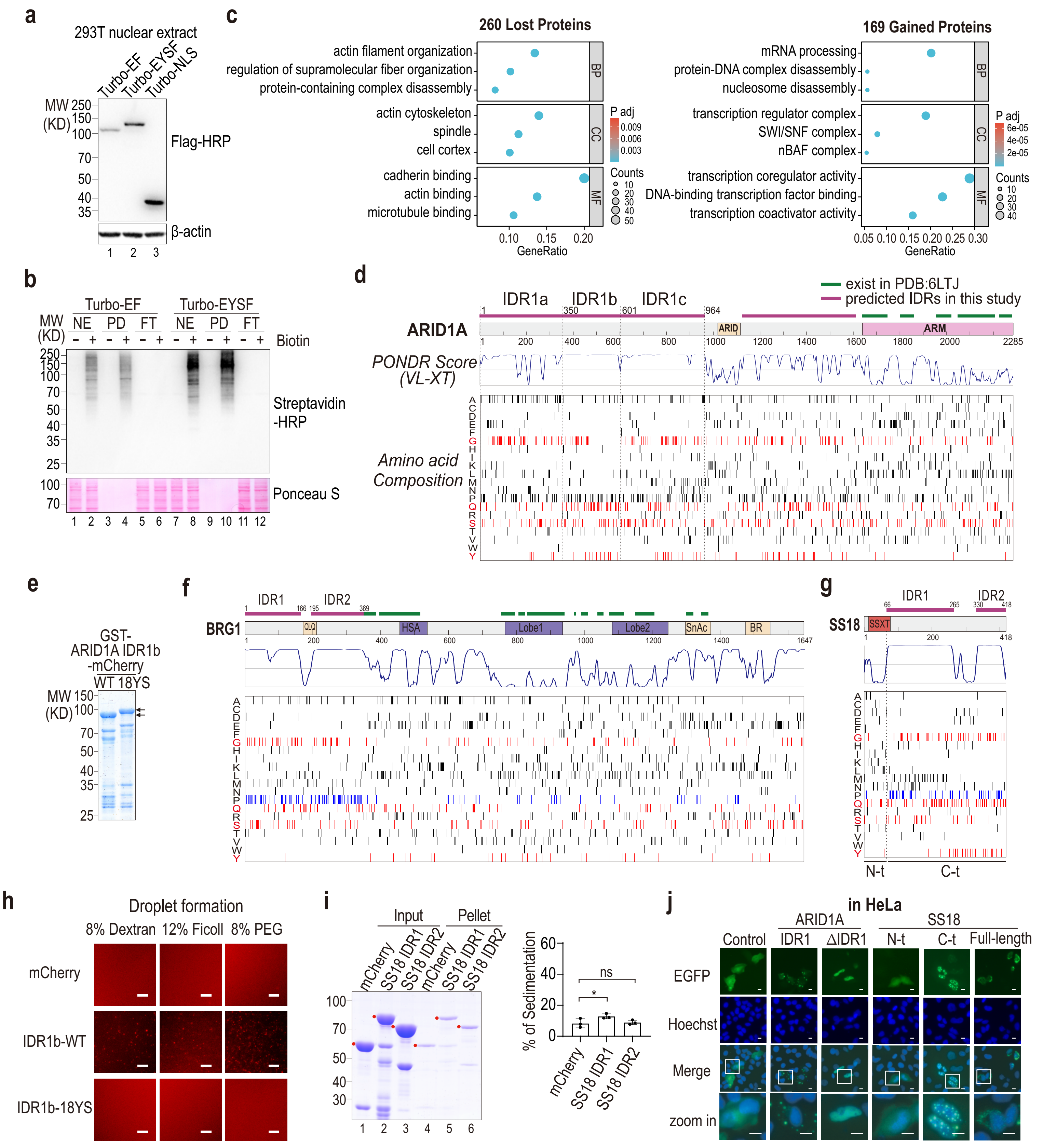

a
293T nuclear extract
Turbo-EF
Turbo-EYSF
Turbo-NLS
MW (KD)
Flag-HRP
β-actin
c
260 Lost Proteins
actin filament organization
regulation of supramolecular fiber organization
protein-containing complex disassembly
actin cytoskeleton
spindle
cell cortex
cadherin binding
actin binding
microtubule binding
BP
CC
MF
P adj
Counts
GeneRatio
169 Gained Proteins
mRNA processing
protein-DNA complex disassembly
nucleosome disassembly
transcription regulator complex
SWI/SNF complex
nBAF complex
transcription coregulator activity
DNA-binding transcription factor binding
transcription coactivator activity
b
Turbo-EF
Turbo-EYSF
NE PD FT NE PD FT
Biotin
Streptavidin -HRP
Ponceau S
d
exist in PDB:6LTJ
predicted IDRs in this study
IDR1a IDR1b IDR1c
ARID1A
ARID
ARM
PONDR Score (VL-XT)
Amino acid Composition
e
GST-ARID1A IDR1b -mCherry
WT 18YS
f
IDR1 IDR2
BRG1
QLQ
HSA
Lobe1
Lobe2
SnAc
BR
g
IDR1 IDR2
SS18
SSXT
N-t C-t
h
Droplet formation
8% Dextran 12% Ficoll 8% PEG
mCherry
IDR1b-WT
IDR1b-18YS
i
Input Pellet
mCherry SS18 IDR1 SS18 IDR2 mCherry SS18 IDR1 SS18 IDR2
% of Sedimentation
ns
*
j
in HeLa
Control
ARID1A
IDR1
ΔIDR1
SS18
N-t
C-t
Full-length
EGFP
Hoechst
Merge
zoom in

Figure S2

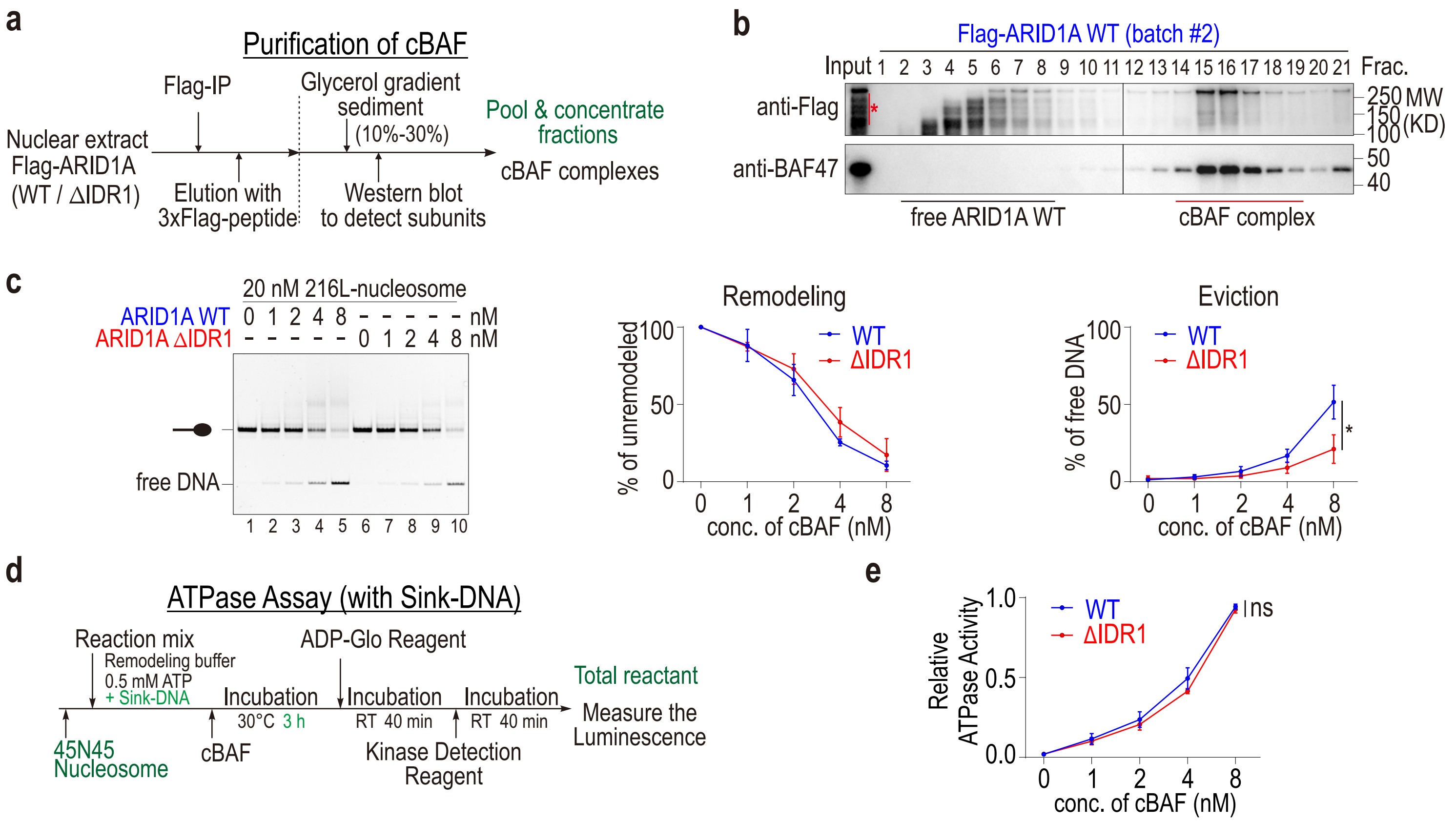

a
Purification of cBAF
Flag-IP
Glycerol gradient sediment
(10%-30%)
Pool & concentrate fractions
Nuclear extract Flag-ARID1A (WT / ΔIDR1)
Elution with 3xFlag-peptide
Western blot to detect subunits
cBAF complexes
b
Flag-ARID1A WT (batch #2)
Input 1 2 3 4 5 6 7 8 9 10 11 12 13 14 15 16 17 18 19 20 21 Frac.
anti-Flag
anti-BAF47
250 MW
150 (KD)
100
50
40
free ARID1A WT
cBAF complex
c
20 nM 216L-nucleosome
ARID1A WT 0 1 2 4 8 - - - - - nM
ARID1A ΔIDR1 - - - - - 0 1 2 4 8 nM
free DNA
1 2 3 4 5 6 7 8 9 10
Remodeling
% of unremodeled
WT
ΔIDR1
conc. of cBAF (nM)
Eviction
% of free DNA
d
ATPase Assay (with Sink-DNA)
Reaction mix
Remodeling buffer
0.5 mM ATP
+ Sink-DNA
ADP-Glo Reagent
Incubation
30°C 3 h
Incubation
RT 40 min
Incubation
RT 40 min
Total reactant
Measure the Luminescence
45N45 Nucleosome
cBAF
Kinase Detection Reagent
e
Relative ATPase Activity
ns
conc. of cBAF (nM)

Figure S3

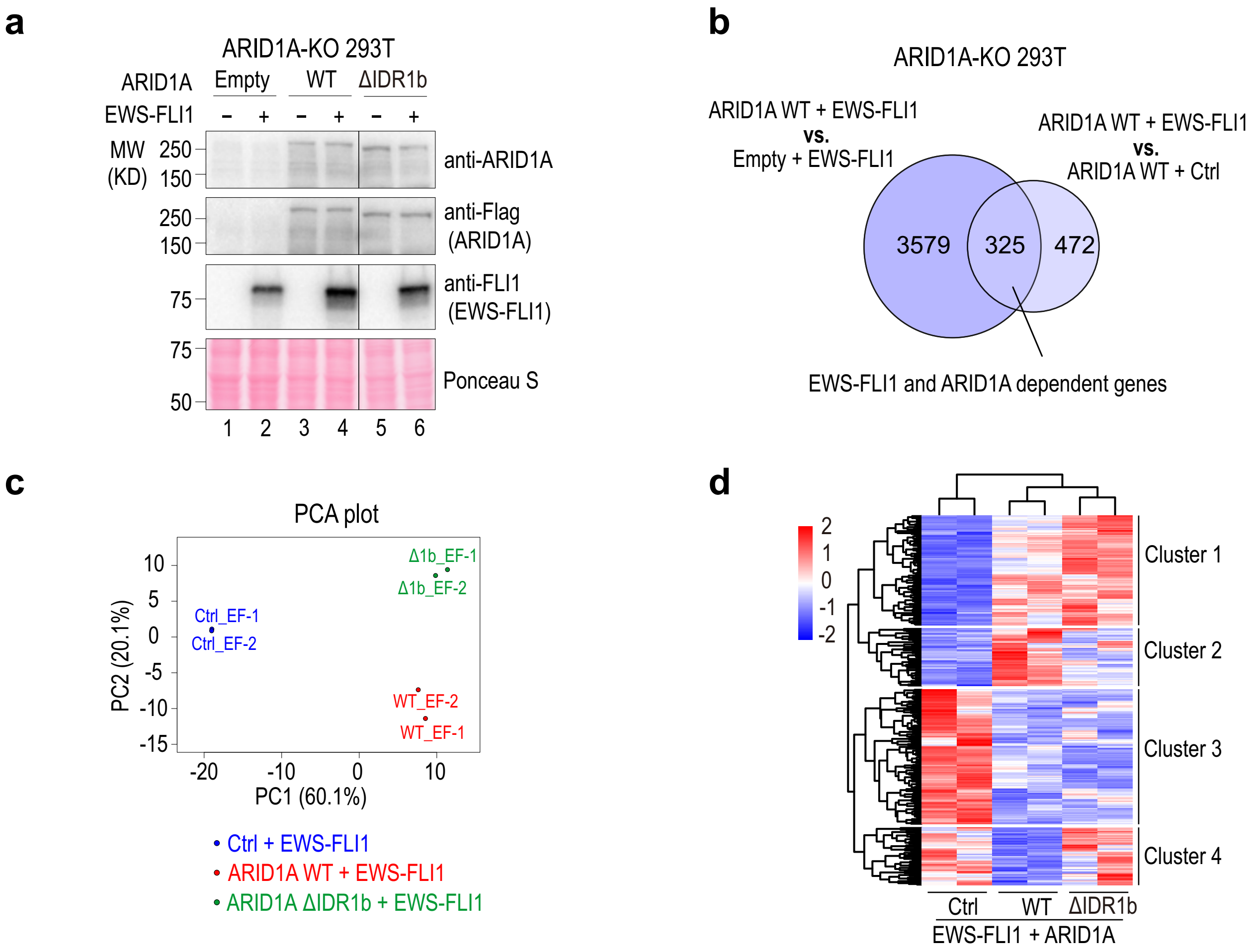
a
ARID1A-KO 293T
ARID1A Empty WT ΔIDR1b
EWS-FLI1 – + – + – +
MW (KD)
250
150
anti-ARID1A
250
150
anti-Flag (ARID1A)
75
anti-FLI1 (EWS-FLI1)
75
50
Ponceau S
1 2 3 4 5 6
b
ARID1A-KO 293T
ARID1A WT + EWS-FLI1 vs. Empty + EWS-FLI1
ARID1A WT + EWS-FLI1 vs. ARID1A WT + Ctrl
3579
325
472
EWS-FLI1 and ARID1A dependent genes
c
PCA plot
Δ1b_EF-1
Δ1b_EF-2
Ctrl_EF-1
Ctrl_EF-2
WT_EF-2
WT_EF-1
PC2 (20.1%)
10
5
0
-5
-10
-15
-20
-10
0
10
PC1 (60.1%)
• Ctrl + EWS-FLI1
• ARID1A WT + EWS-FLI1
• ARID1A ΔIDR1b + EWS-FLI1
d
2
1
0
-1
-2
Cluster 1
Cluster 2
Cluster 3
Cluster 4
Ctrl WT ΔIDR1b
EWS-FLI1 + ARID1A
